# Acceptance and impact of Nirsevimab and the RSVpreF vaccine following implementation in Austria

**DOI:** 10.3389/fpubh.2025.1686581

**Published:** 2025-10-20

**Authors:** Michaela Höck, Wegene Borena, Jürgen Brunner, Karina Wechselberger, Johanna Scheiring, Elisabeth Ralser, Ulrike Pupp Peglow, Peter Wöckinger, Elisabeth D’Costa, Verena Kaiser, Klaus Kapelari, Gisa Gerold, Thomas Müller, Ursula Kiechl-Kohlendorfer, Elke Griesmaier

**Affiliations:** ^1^Department of Pediatrics II, Medical University of Innsbruck, Innsbruck, Austria; ^2^Department of Hygiene, Microbiology and Virology, Institute of Virology, Medical University of Innsbruck, Innsbruck, Austria; ^3^Department of Pediatrics I, Medical University of Innsbruck, Innsbruck, Austria; ^4^Department of Gynecology and Obstetrics, Medical University of Innsbruck, Innsbruck, Austria

**Keywords:** nirsevimab, RSV, newborn, RSVpreF vaccine, acceptance

## Abstract

**Background:**

Since summer 2024, passive immunization with nirsevimab (Beyfortus^®^) has been recommended for all infants in Austria to prevent severe respiratory syncytial virus (RSV) infection. Maternal vaccination with RSVpreF (Abrysvo^®^), which provides transplacental protection, became available in autumn 2023. The expected public health benefits of these preventive strategies depend largely on widespread acceptance; however, real-world data from Austria are unavailable.

**Objective:**

This study aimed to assess the acceptance and impact of RSV immunization strategies during the 2024/2025 season in Tyrol, Austria.

**Methods:**

A retrospective study was conducted analyzing all live births at three Tyrolean maternity wards (Innsbruck, Hall, and Schwaz) from 5 December 2024 to 15 April 2025. Immunization rates were analyzed, and RSV-related hospitalization frequency and duration were compared to pre-pandemic seasons.

**Results:**

Of 1,156 newborns, 57% received nirsevimab and 12% were protected by maternal RSVpreF protection, resulting in an overall coverage of almost 70%. RSV-related hospitalizations for infants under 1 year of age significantly decreased from 151 in pre-pandemic seasons to 47 in the post-nirsevimab season (*p* = 0.018). During the post-nirsevimab season, the median age at hospital admission was significantly higher (*p* < 0.001), and the length of stay was shorter (*p* = 0.031). Importantly, none of the hospitalized infants received nirsevimab, and only one was born to a vaccinated mother.

**Conclusion:**

Our findings highlight the positive impact of both RSV immunization strategies—nirsevimab and RSVpreF vaccine—while underscoring the need to enhance public awareness and education to improve immunization rates. Future immunization programs must be strengthened to provide better protection for the pediatric population and reduce RSV-associated morbidity in early life.

## Introduction

Respiratory syncytial virus (RSV) is the leading cause of bronchiolitis and hospitalizations in infants (1), with peak incidence between the ages of 3 and 6 months and seasonal activity from November to April (2). Despite limited antigenic variation, immunity against RSV is short-lived, and reinfections can occur throughout life, with the first infection often being the most severe (3). In Austria, RSV places a significant seasonal burden on pediatric healthcare, but treatment options are limited to supportive care, underscoring the need for effective preventive measures (4). For over 25 years, palivizumab (Synagis^®^, AstraZeneca), a monoclonal antibody targeting the F-glycoprotein of the virus, was the only prophylaxis of RSV-related lower respiratory tract infections (LRTIs), limited to high-risk infants due to monthly dosing and high costs (5, 6). In October 2022, the European Medicine Agency approved nirsevimab (Beyfortus^®^, Sanofi-Aventis), a single-dose, long-acting monoclonal antibody, and demonstrated its effectiveness in preventing RSV-related LRTIs and reducing hospital admissions by at least 70 to 90% in clinical trials and real-world settings (7, 8). This new monoclonal antibody targets the prefusion conformation of the RSV fusion (F) protein at a highly conserved antigen site, neutralizing a broad panel of RSV A and B viral subgroups (9).

A national campaign launched in December 2024 has provided it free of charge, prioritizing administration before maternity ward discharge with a recommended dose of 50 mg for those under 5 kg and 100 mg for those weighing more than 5 kg at the time of immunization. In parallel, seasonal administration of the bivalent recombinant stabilized prefusion F protein subunit vaccine (RSVpreF, Abrysvo^®^; Pfizer) at a single dose of 120 μg was available for pregnant women between 32 and 36 weeks of gestation to enhance neonatal protection through transplacental antibody transfer (10). These new RSV-preventing strategies are expected to have a strong impact on the burden of medically attended RSV-associated acute respiratory illness (ARI) among young children in Austria. While clinical trials have shown promising efficacy for nirsevimab and the RSVpreF vaccine (11–13), real-world data from Austria are lacking. This study, therefore, aims to assess the acceptance and impact of these RSV immunization strategies during the 2024/2025 season in Tyrol, Austria.

## Materials and methods

### Study design

The study was reported in accordance with the Strengthening the Reporting of Observational Studies in Epidemiology (STROBE) guidelines for observational studies. The analysis was conducted at the maternal ward in the University Hospital Innsbruck—the tertiary care centre in Western Austria—and two peripheral primary care maternity wards in Hall and Schwaz. Immunization data were systematically collected between 5 December 2024 and 15 April 2025. Demographic and clinical data from electronic medical records were used to compare the epidemiology and burden of medically attended RSV-associated ARI in children during the 2024/2025 RSV season with previous seasons, which included two pre-pandemic seasons (2018–2019), the pandemic season (2020–2022), and the transmission seasons (2023–2024). The typical RSV season lasts from October to March. To ensure comprehensive capture of all cases within a given season, the observation period was extended to include 3 months prior to and 3 months after the defined season, resulting in a study period from July to June. Because RSV transmission patterns were disrupted during and after the COVID-19 pandemic, the RSV season after implementation of nirsevimab was compared to pre-pandemic seasons.

### Acceptance

Acceptance was assessed based on documented immunization rates for both nirsevimab and the RSVpreF vaccine across all three maternity wards (University Hospital Innsbruck, state Hospital Hall, and district Hospital Schwaz—both care level I) from 5 December 2024 to 15 April 2025. This period corresponds to the RSV season, which officially ended on April 15, along with the conclusion of the free immunization program. Maternal RSV vaccination status was determined from birth records and prenatal care documentation.

### Impact

The impact was evaluated based on the numbers and length of hospitalization due to RSV infection. During the study period, ICD10 diagnosis codes were used to identify cases of RSV-associated hospitalizations (J12.1; J21.0; J20.5; B97.4). The University Hospital of Innsbruck has the largest pediatric department in Tyrol and serves as the referral center for the most advanced neonatal and pediatric care. It manages a large proportion of all births in southwestern Austria, with most children requiring rehospitalization being admitted here. Inclusion criteria comprised hospitalization due to respiratory symptoms caused by RSV infection, confirmed by a positive antigen or polymerase chain reaction (PCR) test, and documented in the medical chart.

### Ethics approval

The study was conducted in compliance with the Declaration of Helsinki and with the approval of the Ethics Committee of the Medical University of Innsbruck (EC No. 1013/2023).

### Laboratory methods

Among children presenting with symptoms of respiratory tract infection, RSV status was determined using mid-turbinate nasal or oropharyngeal specimens, analyzed by either rapid antigen testing or institution-specific in-house PCR assays. Patient samples were processed within 24 h using an automated RNA extraction system (EMAG, Biomerieux), followed by RSV genotyping via RealStar RSV-RT-PCR to distinguish RSV A and B (Altona, Germany). Samples with a CT value below 30 were selected for further analysis. An F protein targeted Sanger sequencing was conducted. Amplified F-gene products were sequenced and aligned with reference strains and previous Austrian sequences (GISAID) to detect mutations in nirsevimab-relevant epitopes.

### Statistical analysis

A retrospective data analysis was performed using SPSS, version 29.0 for Windows (IBM Corp., Chicago, IL, USA). Descriptive statistics were used to characterize the individual variables and to determine the distribution of data, using the Shapiro–Wilk test. Values are expressed as numbers (frequencies, %), mean with standard deviation (±SD), and median with interquartile range (IQR). The Mann–Whitney *U-*test, Student’s *t*-test, and the χ2 test were used where appropriate. A *p*-value of <0.05 was considered statistically significant.

## Results

During the study period (5 December 2024 to 15 April 2025), a total of 1,156 neonates were born across the three participating maternity wards (Innsbruck *n* = 706, Hall *n* = 279, and Schwaz *n* = 171), of them 559 were male newborns (48%).

### Acceptance

At the University Hospital of Innsbruck, 368 out of 706 newborns (55%) received nirsevimab, and 60 mothers (8%) were vaccinated with the RSVpreF vaccine during pregnancy. In the district hospital Schwaz, 105 out of 171 infants (61%) were given nirsevimab, and 23 mothers (13%) were vaccinated with the RSVpreF vaccine. In the state hospital Hall, 279 births were recorded, with 101 newborns (36%) receiving nirsevimab and 32 mothers (11%) vaccinated during pregnancy.

**Figure 1:**
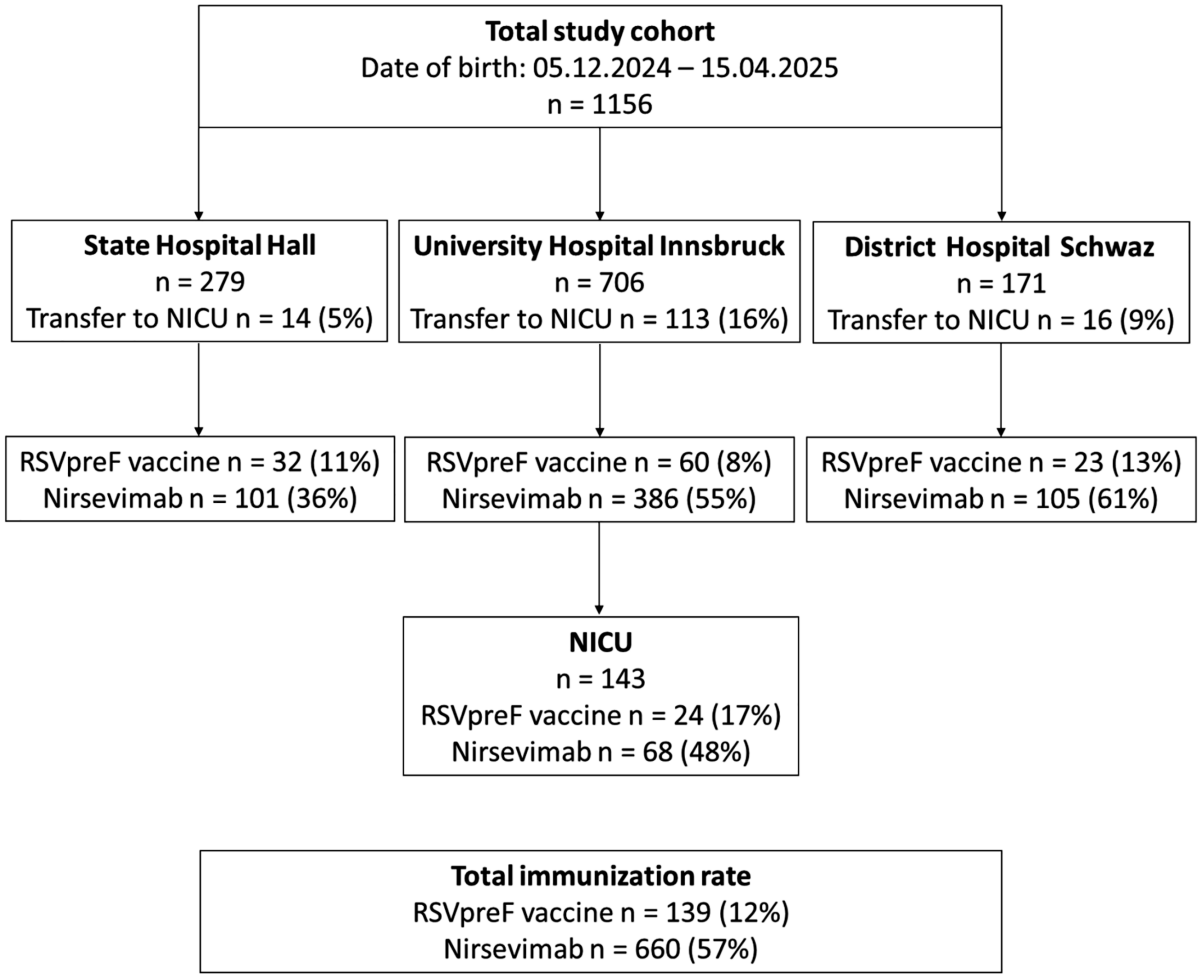
Flow chart of immunization with nirsevimab and RSVpreF vaccine.

A total of 143 infants (12%) were admitted to the neonatal intensive care unit (NICU) due to prematurity or neonatal conditions such as congenital malformations, respiratory distress syndrome, pulmonary hypertension, perinatal asphyxia, sepsis, or hypoglycemia before nirsevimab immunization. Transfers to the NICU were documented to minimize potential bias and are illustrated in detail in Figure 1.

In total, 88 infants (57 preterm and 31 term-born) were immunized with nirsevimab in the NICU, notably including 23 born before 5th December. These patients had a mean birth weight of 2,381 g (range: 730–4,200 g) and a mean gestational age of 35 + 2 weeks (range: 24 + 2 to 42 + 0 weeks). Additionally, 27 infants were born to mothers who had received the RSVpreF vaccine. Three preterm infants received palivizumab.

In the study cohort, 660 newborns (57%) were discharged with protection against RSV via nirsevimab and 139 (12%) via maternal RSV vaccination, yielding a total prophylactic coverage of almost 70%. Immunization rates varied significantly between the three hospitals (*p* < 0.001), with nirsevimab rates ranging from 36% in Hall to 61% in Schwaz, and RSVpreF vaccination rates ranging from 8% in Innsbruck to 13% in Schwaz. An overview of the immunization rates is provided in Figure 1.

**Figure 2:**
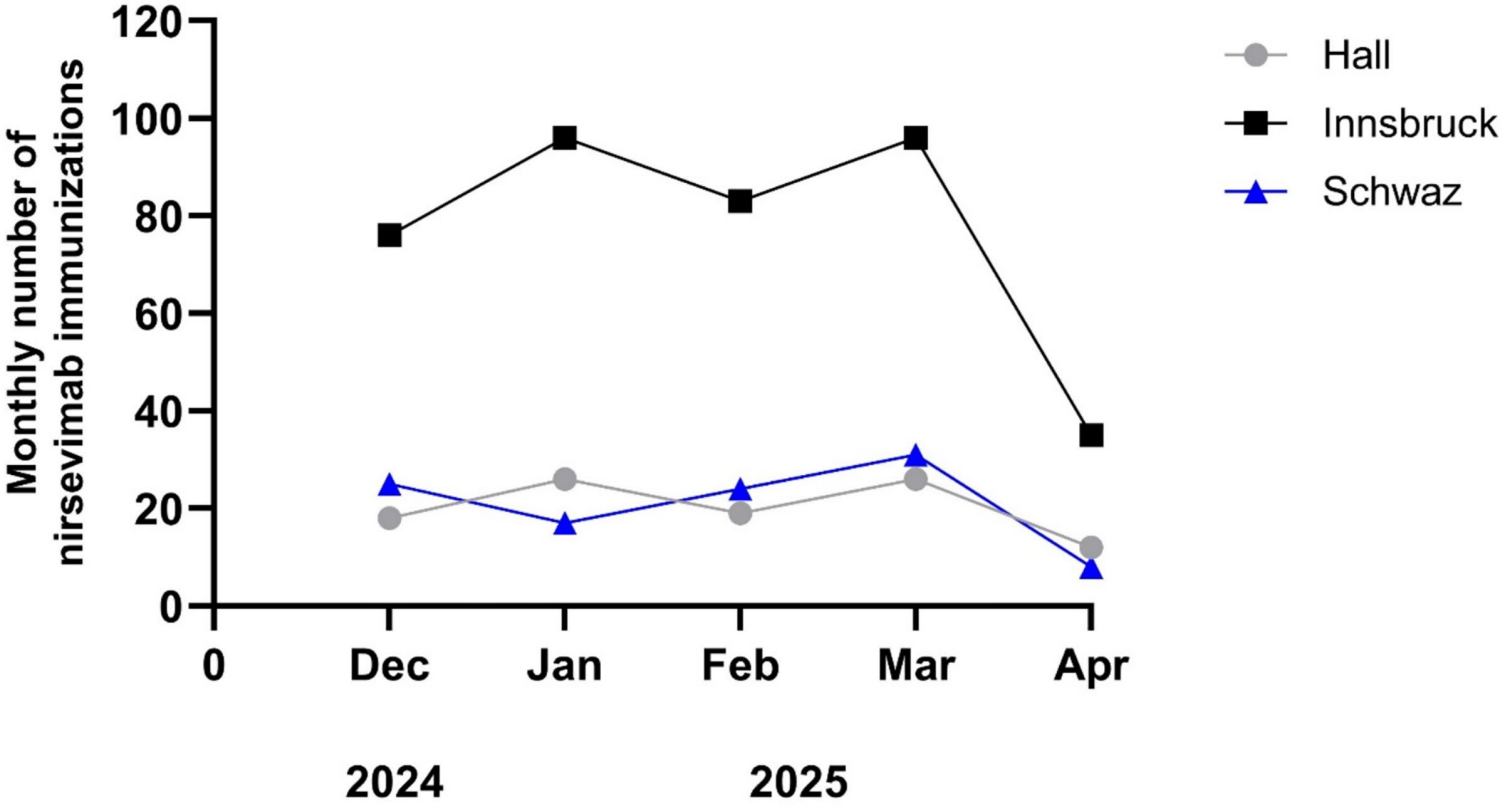
Immunization rates of nirsevimab on the three maternal wards (Innsbruck, Hall, and Schwaz).

The timing of immunizations showed significant variation, with peaks in January (*n* = 139, 23%) and March (n = 153, 25%), and the lowest rate recorded in April (*n* = 55, 9%), as illustrated in Figure 2. Similarly, the highest number of NICU immunizations also occurred during these 2 months, with January accounting for 23 (34%) and March for 25 (37%) immunizations.

### Impact

**Figure 3:**
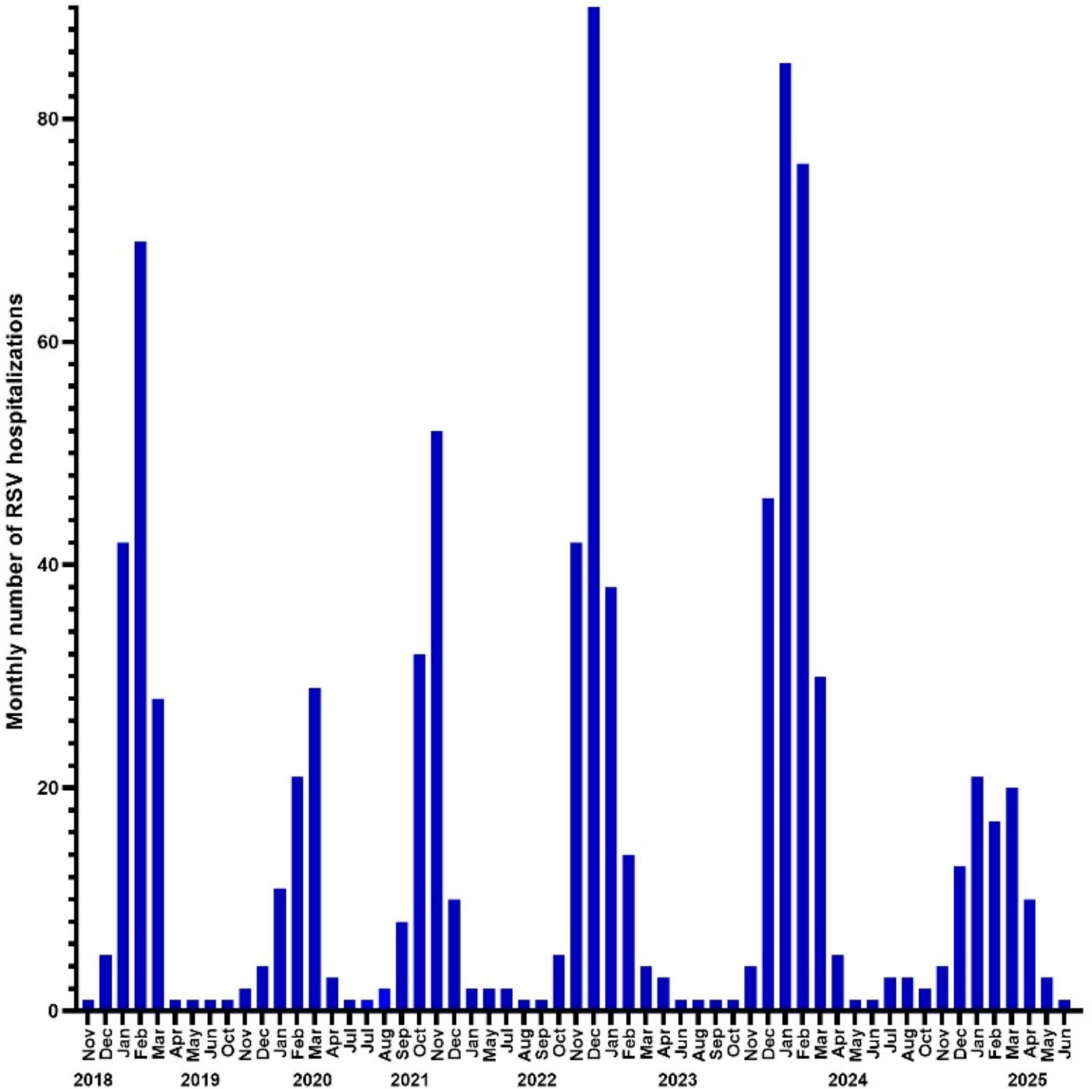
Number of RSV-hospitalized infants from 2018 to 2025.

Between 2018 and 2025, 882 patients under 18 years of age were hospitalized due to RSV infection, of whom 572 (65%) were infants below age 1 year. The 2024/2025 RSV season showed a similar seasonal trend to the previous year, with cases beginning to rise in December and peaking in January. Compared to pre-pandemic seasons, there was an apparent decline in RSV-related hospitalizations during the 2024/2025 season—similar to the reductions observed during the COVID-19 pandemic (Figure 3). This decline was particularly significant among infants under 1 year of age (151 vs. 47; *p* = 0.018).

Table 1 summarizes hospitalizations across RSV seasons since 2018, detailing patient age, gender, and length of hospital stay.

**Table 1:** Children hospitalized with RSV-associated acute respiratory illness.

Variable	Total	Pre-pandemic seasons	Pandemic seasons	Transition seasons	Nirsevimab
		2018–2019	2020–2021	2022–2024	5th Dec 2025
Total hospitalizations, *n* (%)	882	219 (24.8)	110 (12.5)	468 (53.1)	85 (9.6)
Age [d], median (IQR)	190 (66; 586)	158 (53; 440)	121 (45; 420)	205 (73; 640)	269 (116; 964)
Age < 1 year, *n* (%)	572	151 (69)	78 (71)	296 (63)	47 (55)
Male sex, *n* (%)	503 (57)	114 (52)	66 (60)	274 (59)	49 (58)
Length of stay [d], median (IQR)	3 (2; 5)	3 (2; 5)	4 (3; 7)	3 (2; 5)	2 (1; 5)

### RSV-associated deaths

Since 2018, five RSV-associated deaths have been reported in the pediatric department of the university hospital Innsbruck, all involving children with severe underlying conditions. In 2021, a 16-month-old patient with mumps parotitis experienced complications from acute liver failure. In 2023, a 16-month-old patient with a genetic syndrome involving a deletion in the 1q23.3 to q25.1 region. In 2024, a 14-year-old boy with an SMARCB1-deficient malignant tumor of the left orbit, staged T4N0M0. Additionally, in 2024, a 3-year-old boy had myotubular myopathy caused by an MTM1 gene mutation. There was also a 15-year-old boy with developmental and epileptic encephalopathy due to terminal deletion of Xp22.33 and terminal gain of Xq28.

### Hospitalizations

Between 5 December 2024 and 9 June 2025, a total of 85 patients (including 9 preterm infants with a gestational age range of 24 + 0 to 36 + 4 weeks, and 76 term-born infants) were hospitalized due to severe lower respiratory tract infections (LRTI) caused by RSV. Diagnosis of RSV was primarily confirmed by PCR or rapid antigen testing. The median length of hospital stay was 2 days (1; 5), with a median patient age of 269 (116; 964) days. Seven patients (8%) required admission to the pediatric intensive care unit. Supplemental oxygen was administered to 42 patients. Seven patients required non-invasive respiratory support, and two were intubated.

**Figure 4:**
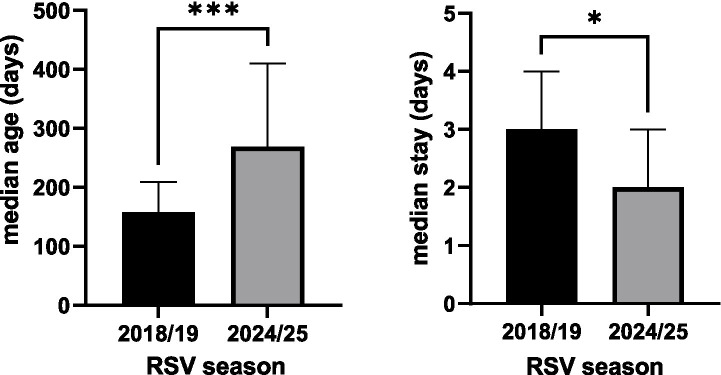
Median age at admission and median hospital stay, with *p* < 0.001 marked with *** and *p* < 0.05 marked with *.

In the post-nirsevimab RSV season, the median age at admission was significantly higher (*p* < 0.001), while the median length of hospital stay was significantly shorter (*p* = 0.031) compared to the pre-pandemic period, as shown in Figure 4.

### Immunization status

Among all patients hospitalized since the implementation of the new RSV prevention strategies, one term-born infant was hospitalized at the age of 2.5 months for 2 days with RSV bronchiolitis, although no respiratory support was needed. The infant’s mother had received Abrysvo® 14 days before delivery.

A term-born infant with congenital diaphragmatic hernia who had received nirsevimab was diagnosed with bronchiolitis at 4 months of age, requiring a 3-day hospitalization. At the time of discharge, the results of the respiratory panel were still pending. Subsequent testing did not detect RSV; instead, nucleic acids specific to rhinovirus were identified in the respiratory specimen.

Three former preterm infants had been immunized with palivizumab; however, their RSV infections occurred at 4.5, 1.6, and 2.9 years of age, respectively—well beyond the period during which protection from the antibody would be expected.

The analysis of F protein sequences from 14 samples collected during the 2024/2025 RSV season did not identify any relevant mutations at the Nirsevimab binding site (previously unreported). The subgroup analyses for children hospitalized with RSV-associated ARI since the implementation of nirsevimab are presented in Table 2.

**Table 2:** Subgroup analyses for children hospitalized with RSV-associated ARI: from December 2024 to June 2025 * *n* = 14.

Variable	Nirsevimab 2025
Total hospitalizations, *n*	85
Re-hospitalizations, *n* (%)	5 (6)
Admissions to the pediatric intensive care unit, *n* (%)	7 (8)
Death, *n* (%)	1 (1)
Preterm birth (gestational age < 37 weeks), *n* (%)	9 (11)
Term birth (gestational age ≥ 37 weeks), *n* (%)	76 (89)
Male sex, *n* (%)	49 (58)
Supplemental oxygen receipt, *n* (%)	42 (49)
Non-invasive respiratory support, *n* (%)	7 (8)
Mechanical ventilation, *n* (%)	2 (2)
Heart rate [bpm], median (IQR)	156 (144; 176)
Transcutaneous oxygen saturation [%], median (IQR)	92 (88; 99)
Temperature [°C], median (IQR)	37,8 (37,3; 38,6)
Respiratory rate [pm], median (IQR)	55 (40; 64)
Blood pressure systolic [mmHg], median (IQR)	99 (93; 108)
Blood pressure diastolic [mmHg], median (IQR)	65 (58; 73)
RSV test: Antigen, PCR; not detected, *n* (%)	53 (62), 30 (35), 2 (2)
Immunization, *n* (%)	5 (6)
RSV Genotyp* (A; B)	12; 2
Mutations at the Nirsevimab binding site*	2 (K65R)

## Discussion

RSV remains one of the leading causes of LRTIs in infants and young children, placing a considerable burden on healthcare systems each winter season (14–16). The introduction of two new RSV immunization strategies—nirsevimab for all neonates and infants, and maternal vaccination with the RSVpreF vaccine—marks a major advancement in pediatric infectious disease prevention (11, 12).

This study provides the first real-world report in Austria following the implementation of these new RSV immunization strategies. In the 2024/2025 RSV season, 680 newborns (58%)—including 23 preterm infants born before 5th December 5—were discharged with protection against severe RSV infection via nirsevimab, and 142 (12%) via maternal RSVpreF vaccination. Nonetheless, the uptake of both immunization strategies differed markedly among the three maternal wards. Nirsevimab coverage ranged from 36% in Hall to 55% in Innsbruck and 61% in Schwaz. A similar pattern was observed for the maternal RSVpreF vaccine, with uptake rates of 11% in Hall, 8% in Innsbruck, and 13% in Schwaz. These differences likely reflect variations in local implementation logistics, levels of parental education, and the degree of healthcare provider engagement. We found low uptake of the maternal RSV vaccination, although it became available in September 2023, which highlights the need to better integrate this intervention into routine prenatal care and to strengthen awareness among pregnant women and obstetric healthcare providers. Moreover, nirsevimab is included in the publicly funded national immunization program, whereas the RSVpreF vaccine is not covered and must be purchased privately. Martin et al. found that parents of immunized infants were more likely to have a university education (60.2% vs. 36.1%) and to be vaccinated against influenza (49.9% vs. 21.5%) compared to those of non-immunized infants. Awareness of RSV was higher among parents of immunized infants (73.8%), with 70.1% feeling well-informed, whereas 59.8% of non-immunized parents were unaware of RSV and reported feeling poorly informed. The majority of parents identified pregnancy as the optimal time to receive immunization information. Preferences for immunization strategies differed: 32.4% of immunized parents favored infant vaccination, while 60.3% of non-immunized parents preferred maternal vaccination. Despite some safety-related hesitancy, high parental acceptance and satisfaction with nirsevimab were reported during the 2023–2024 season in the Murcia region (17). Consistent with previous data, no serious adverse events were observed in our study; the most common side effects reported were mild, such as rash and injection-site irritation. These findings support the favorable safety profile of nirsevimab up to 360 days post-administration, regardless of gestational age or comorbidities (18–20). The effective reduction of RSV-associated hospitalizations and the economic burden was shown by several studies (16, 21).

The introduction of passive immunization with nirsevimab for all neonates and infants, combined with maternal vaccination using RSVpreF, has led to a significant reduction in RSV-related hospitalizations among patients under 1 year of age. This decline parallels the decrease observed during the COVID-19 pandemic, when public health measures such as social distancing disrupted RSV transmission. While RSV circulation was markedly altered during the pandemic, our data indicate a return to typical seasonal patterns, with infection rising in December and peaking in January, consistent with other reports (22). This re-emergence of predictable patterns reinforces the importance of timely and widespread immunization prior to peak RSV activity. Importantly, this reduction in hospital burden occurred despite immunization rates of 57% for nirsevimab and even lower rates of maternal vaccination with the RSVpreF vaccine (12%). In our study, we observed a shift toward older age at the time of infection and a reduced duration of hospitalization. This positive real-world impact observed in our study aligns with post-licensure data from other European countries, which have reported risk reductions exceeding 80% (8, 23–27). Notably, a term-born infant aged 2.5 months was hospitalized with confirmed RSV bronchiolitis, diagnosed by rapid antigen testing. The infant’s mother had received RSVpreF vaccination 14 days prior to delivery, indicating that while maternal immunization may attenuate disease severity, breakthrough infections can still occur, though in this case without requiring respiratory support. Conversely, another term-born infant with congenital diaphragmatic hernia who had received nirsevimab developed bronchiolitis at 4 months of age, necessitating a brief hospital stay. Interestingly, RSV was not detected in this case; instead, rhinovirus-specific nucleic acids were identified, suggesting that despite prophylaxis, infants remain susceptible to other viral pathogens causing similar clinical symptoms. These findings highlight the strong protective effect of the new RSV immunization strategies, while also underscoring the complexity of RSV prevention and the need for ongoing surveillance to better understand the spectrum of viral etiologies and the effectiveness of current prophylactic measures. Ernst et al. compared two consecutive seasons (2022/23 and 2023/24) and also showed the impact of nirsevimab in mitigating severe RSV-disease, following a national implementation in Luxembourg, with an immunisation coverage of up to 84% among neonates (26). This study is supported by a recent model simulation by Du et al., demonstrating the potential of RSVpreF vaccines for public health, but assuming high vaccination uptake rates (28).

Sustained substantial protection provided by nirsevimab will also depend on the ongoing genetic stability of the targeted epitope. Prior molecular studies have demonstrated that the RSV F protein, particularly the epitope targeted by nirsevimab (site Ø), exhibits low genetic diversity (29). Nevertheless, with the widespread and increasing use of monoclonal antibodies in the current and forthcoming seasons, there is a plausible concern that it may exert selective pressure on the circulating virus, potentially promoting the emergence and spread of escape variants. Although our retrospective analysis of F protein sequences from 14 samples collected during the 2024/25 RSV season did not identify any mutations associated with nirsevimab resistance, ongoing comprehensive genomic surveillance of circulating RSV remains crucial, particularly among hospitalized pediatric populations (30).

## Limitation

Several limitations should be noted. The availability of nirsevimab only after the onset of the RSV season limited the window of prophylactic protection for some infants, potentially reducing the overall impact observed. Additionally, incomplete vaccine uptake and delayed immunization may have constrained the full impact of the campaign. Moreover, while hospital data were robust, cases managed in other healthcare settings or those with milder disease may be underrepresented, as data from smaller Tyrolean hospitals were not included.

## Conclusion

The introduction of nirsevimab and maternal RSVpreF vaccination in Tyrol, Austria, represents a major step forward in preventing severe RSV disease in infants. This real-world evaluation confirms their positive impact on reducing RSV-related hospitalizations. However, limited product availability and suboptimal coverage during the first season hindered their full potential. To maximize public health impact, efforts must focus on improving awareness among parents and healthcare providers, overcoming logistical challenges, and integrating maternal vaccination into routine prenatal care. Strengthened collaboration between hospitals, pediatricians, and obstetric services will be crucial to ensure timely, broad, and equitable protection for all infants.

## Data Availability

The raw data supporting the conclusions of this article will be made available by the authors, without undue reservation.
